# Effects of Bacterial Lysates and Metabolites on Collagen Homeostasis in TNF-α-Challenged Human Dermal Fibroblasts

**DOI:** 10.3390/microorganisms11061465

**Published:** 2023-05-31

**Authors:** Laura Huuskonen, Heli Anglenius, Ilmari Ahonen, Kirsti Tiihonen

**Affiliations:** 1 IFF Health and Biosciences, Sokeritehtaantie 20, 02460 Kantvik, Finland; heli.anglenius@iff.com (H.A.); kirsti.tiihonen@iff.com (K.T.); 2 Vincit Oyj, Helsinginkatu 15, 20500 Turku, Finland; ahosen.ilmari@gmail.com

**Keywords:** probiotic, postbiotic, human dermal fibroblast, collagen, matrix metalloproteinase, skin aging, cytokines

## Abstract

During skin aging, the production of extracellular matrix (ECM) proteins, such as type I collagen, decreases and the synthesis of ECM-degrading matrix metalloproteinases (MMPs) rises, leading to an imbalance in homeostasis and to wrinkle formation. In this study, we examined the effects of bacterial lysates and metabolites from three bifidobacteria and five lactobacilli on collagen homeostasis in human dermal fibroblasts during challenge with tumor necrosis factor alpha (TNF-α), modeling an inflammatory condition that damages the skin’s structure. Antiaging properties were measured, based on fibroblast cell viability and confluence, amount of type I pro-collagen, ratio of MMP-1 to type I pro-collagen, cytokines, and growth factors. The TNF-α challenge increased the MMP-1/type I pro-collagen ratio and levels of proinflammatory cytokines, as expected. With the probiotics, differences were clearly dependent on bacterial species, strain, and form. In general, the lysates elicited less pronounced responses in the biomarkers. Of all strains, the *Bifidobacterium animalis* ssp. *lactis* strains Bl-04 and B420 best maintained type I pro-collagen production and the MMP-1/collagen type I ratio under no-challenge and challenge conditions. Metabolites that were produced by bifidobacteria, but not their lysates, reduced several proinflammatory cytokines (IL-6, IL-8, and TNF-α) during the challenge, whereas those from lactobacilli did not. These results indicate that *B. animalis* ssp. *lactis*-produced metabolites, especially those of strains Bl-04 and B420, could support collagen homeostasis in the skin.

## 1. Introduction

Skin aging is a complex process that is influenced by a combination of intrinsic and extrinsic factors. In the dermis, fibroblasts secrete extracellular matrix (ECM) components, the most significant of which is type I collagen. In aging skin, the increase in senescent cells can impede the ability to synthesize collagen. Decreases in the number of dermal fibroblasts, reduced ECM protein synthesis, and greater protein degradation can disrupt the structural integrity and cause wrinkle formation [1].

Tumor necrosis factor-α (TNF-α) is a signaling molecule that is released by immune cells, as well as skin cells. Its effects vary between cells, from modulating the cell cycle and proliferation to inducing apoptosis and necrotic cell death. In the skin, TNF-α inhibits collagen synthesis, promotes matrix metalloproteinases (MMPs), and upregulates other proinflammatory cytokines, such as interleukin (IL)-6 and IL-8 [2], which can aggravate such proinflammatory effects. TNF-α overexpression is induced by ultraviolet (UV) radiation [3] and cellular senescence, which increases with age [4]. The term “inflammaging” has been used to describe chronic, low-grade, underlying inflammation—which also occurs in the skin—that can result from lifestyle and environmental factors [5]. The long-term presence of TNF-α increases reactive oxygen species (ROS) levels and causes premature senescence in human dermal fibroblasts [6], leading to a cycle of harmful effects.

Probiotics are defined as “live microorganisms that, when administered in adequate amounts, confer a health benefit on the host” [7]. Postbiotics, derived from living microbes, are considered as “preparation of inanimate microorganisms and/or their components that confers a health benefit on the host” [8]. Thus, postbiotics can be viewed as inactivated microbial cells or cell components, with or without metabolites, that confer specific health benefits [8]. In skin care products, postbiotics are typically used in topical formulations, whereas oral benefits are provided through the gut–skin axis, likely via modulation of microbial activity, gut permeability, and the immune system [9].

Our aim was to screen potential probiotic candidates in vitro that support collagen homeostasis. We simulated in vitro the effects of oral probiotics on skin by applying lysed bacterial fragments or bacterial metabolites (postbiotics) to human dermal fibroblasts (HDFs) in the presence or absence of an inflammatory challenge (TNF-α). The effects on collagen homeostasis were evaluated on the basis of HDF viability and confluence and the levels of type I pro-collagen, MMP-1, cytokines, and growth factors.

## 2. Materials and Methods

### 2.1. HDF Cell Culture

The screening experiments were conducted in normal primary HDFs that were isolated from the skin of a young adult (Thermo Fisher Scientific, Waltham, MA, USA). The cells were maintained in Medium 106 that was supplemented with low serum growth supplement (LSGS) and 1% antibiotic–antimycotic (all reagents for cell culture were purchased from Thermo Fisher Scientific) at 37 °C and 5% CO_2_ in a humidified atmosphere and sub-cultured per the manufacturer’s instructions. The experiments were conducted with cell passages 6–8. Cell viability and confluence were determined in five replicates per treatment, and ELISA was performed with three replicates per treatment.

### 2.2. Bacterial Cell Culture and Sample Preparation

The *Bifidobacterium animalis* ssp. *lactis* strains B420 (DGCC420), Bi-07 (DGCC12895), and Bl-04 (DGCC2908) were cultured anaerobically at 37 °C in *Bifidobacterium* medium No. 58 (DSMZ GmbH, Braunschweig, Germany). *Lactiplantibacillus plantarum* LP12407 (DGCC12407) and LP12418 (DGCC12418), *Lacticaseibacillus paracasei* Lpc-37 (DGCC4981), *Lacticaseibacillus rhamnosus* HN001 (DGCC1460), and *Ligilactobacillus salivarius* Ls-33 (DGCC9868) were cultured anaerobically in de Man, Rogosa, and Sharpe (MRS) broth (Neogen, Lansing, MI, USA) at 37 °C. The bacterial strains were obtained from the Danisco Global Culture Collection (DGCC, Niebüll, Germany).

The bacteria were grown until the exponential growth phase, and their cell densities were measured using an optical density meter (BioPhotometer, Eppendorf, Hamburg, Germany) and by flow cytometry (FACSCalibur, Becton Dickinson, San Jose, CA, USA) per Apajalahti et al. [10]. Bacterial lysate and metabolite samples were prepared per Putaala et al. [11] with few modifications. In brief, bacterial cells and soluble metabolites were separated by centrifugation at 2216× *g* for 5 min at 4 °C. The bacterial pellets were resuspended in Medium 106 supplemented with LSGS and then lysed with Precellys 24 bead beater (Bertin Technologies, Saint-Quentin-en-Yvelines Cedex, France) with a VK01 grinding kit (Bertin Technologies) at 6800 rpm for three cycles of 30 s, with the samples on ice between cycles. Breakdown of the cells was confirmed by light microscopy. The lysed bacterial cells, sterile-filtered on 0.2 μm syringe units (Sartorius, Göttingen, Germany), were administered at a ratio of 10 bacterial cells to one dermal fibroblast, diluted in supplemented Medium 106. The soluble metabolites were diluted to 1% (*v*/*v*) in supplemented Medium 106, and sterile-filtered on 0.2 μm syringe units before being applied to HDFs.

### 2.3. Inflammatory Challenge

HDFs were subjected to an inflammatory challenge with recombinant human TNF-α (Thermo Fisher Scientific) at 20 ng/mL in supplemented Medium 106, with bacterial lysates or metabolites.

### 2.4. Cell Viability and Confluence

Cell viability was evaluated by MTT assay. Briefly, HDFs (1 × 10^4^ cells/well) were plated in 96-well plates (Nunc™ MicroWell™, Thermo Fischer Scientific); after 24 h, 0.1 mL of test samples (bacterial lysates, metabolites, and controls) were applied and incubated for 72 h. Next, HDFs were washed twice carefully with phosphate-buffered saline (PBS) and treated for 3 h with 0.5 mg/mL MTT (thiazolyl blue tetrazolium bromide, Sigma-Aldrich, St. Louis, MO, USA) diluted in Dulbecco’s modified Eagle medium (DMEM) (Thermo Fisher Scientific) at 37 °C and 5% CO_2_ in a humidified atmosphere, after which the solution was replaced with 0.12 mL dimethyl sulfoxide (Sigma-Aldrich). After a 5 min incubation on an orbital shaker, protected from light, 90 μL of lysate was transferred to a 96-well plate, and the absorbance was measured at 570 nm (subtracting the background absorbance of 700 nm) on an EnSight plate reader (Perkin Elmer, Waltham, MA, USA).

Confluence was measured to assess cell morphology-related aspects by determining the area of the well bottom that was covered by the cells. To measure confluence, HDFs were cultured and treated with test samples as described above. After 72 h of incubation with the test samples and two careful washes with PBS, confluence was determined by brightfield imaging on an EnSight plate reader with the accompanying software (Kaleido version 2.0.3058.126).

### 2.5. ELISA

A cell culture method described by Hsu et al. [12] was used to evaluate the biosynthesis of various molecules (see below) by enzyme-linked immunosorbent assays (ELISAs), with some modifications. Briefly, 4 × 10^4^ HDFs/well were plated in 24-well plates (Costar^®^, Corning, NY, USA) and incubated at 37 °C and 5% CO_2_ in a humidified atmosphere for 72 h. Next, 1 mL of test samples were applied to the cells and incubated for an additional 72 h. Human transforming growth factor beta 1 (TGF-β1) (Sigma-Aldrich), diluted to 1 ng/mL in supplemented Medium 106, was used as a positive control for type I pro-collagen synthesis [13]. After incubation, the cell culture medium was collected for ELISA, and the cells were washed once with warm PBS and lysed in CelLytic™ M reagent (Sigma-Aldrich) per the manufacturer’s instructions. The protein concentration of the lysates was measured using the Pierce BCA Protein Assay Kit (Thermo Fisher Scientific) per the manufacturer’s instructions. The ELISA results were normalized to the protein concentration of the corresponding HDF lysate.

#### 2.5.1. Measurements of Type I Pro-Collagen and MMP-1

Type I pro-collagen content in the HDF culture supernatant was analyzed with the Pro-Collagen I α1 kit (R&D Systems, Minneapolis, MN, USA). MMP-1 was measured with the Total MMP-1 kit (R&D Systems), which detects the human active and pro forms of the proteinase. Both kits were used per the manufacturer’s instructions. MMP-1 levels were expressed relative to type I pro-collagen as the MMP-1/type I pro-collagen ratio.

#### 2.5.2. Measurements of Cytokine and Growth Factor Concentrations

Multiplex ELISA was used to analyze the concentration of IL-1α, IL-1β, IL-6, IL-8, IL-10, TNF-α (SP-X Human 6-Plex Array), vascular endothelial growth factor (VEGF), and basic fibroblast growth factor (FGFb) (SP-X Human 2-Plex Array). Singleplex assays were used to determine the concentrations of keratinocyte growth factor (KGF; Simoa^®^ KGF Developer Kit) and TGF-β1 (Simoa^®^ TGFβ1 Developer Kit) in the cell culture supernatants. The multiplex and singleplex ELISA kits were purchased from Quanterix (Billerica, MA, USA) and used per the manufacturer’s instructions.

### 2.6. Statistical Analysis

Although only minimal systematic difference between the successive fibroblast cell passages was observed, the bacterial lysate- and metabolite-induced biomarker values were nonetheless normalized for possible passage effect. This was achieved by first calculating the average differences between the controls originating from different passages and then subtracting this difference from all biomarker data. The magnitude of the passage effect was estimated by the proportion of biomarker variance explained in comparison to the amount attributable to the compound and challenge in an analysis of variance (ANOVA).

Principal components were calculated for the data, wherein each observation consisted of the mean of a biomarker for each combination of probiotic strain, sample type, and challenge. The data were standardized to a mean of 0 and variance of 1 to give each biomarker equal weight. Spearman correlations were calculated between biomarker pairs, and their statistical significance was determined using asymptotic *p*-values.

Pairwise comparisons of all treatments against the corresponding controls were performed separately for each biomarker (lysate and metabolite samples were compared with the respective control HDF passage). The control treatments were medium for the no-challenge data, TNF-α for the challenge data for all biomarkers, and TGF-β1 for differences in type I pro-collagen. The statistical analysis was performed by standard *t*-test if group variances did not differ significantly by Levene’s test; Welch’s *t*-test was used if they did. For cases in which a comparison was not possible due to missing values, the resulting *p*-value was set to 1 and included in subsequent corrections for multiple simultaneous tests. All *p*-values were corrected for false discovery rate (FDR) by the Benjamini–Hochberg method. For all comparisons, *p* < 0.05 was considered to be statistically significant. Effect sizes were reported as fold-changes, which was defined as the ratio of the means of the compared groups. All analyses were performed in R, version 4.2.2.

## 3. Results

The effect of cell passage was assessed by measuring the variability in data between passages, particularly between test samples (lysed bacterial cells, metabolites, and controls) and challenged versus unchallenged samples. In nearly all cases, the effects of the test samples and challenge clearly surpassed the passage effect by several orders of magnitude. The exceptions were FGFb, IL-1β and TGF-β1, for which the passage effect was relatively high (9.2%, 9.1% and 8.3% of the variance, respectively) but smaller than that of the test samples and challenge.

### 3.1. Effects of Systemic Inflammatory Challenge 

The effects of lysed bacteria and bacterial metabolites on HDFs were determined under no-challenge and TNF-α-challenged conditions, the latter of which was used to model systemic inflammation and the effects of external stressors, such as UV radiation. To differentiate the effects of the challenge across all data, we conducted principal component analysis (PCA).

**Figure 1:**
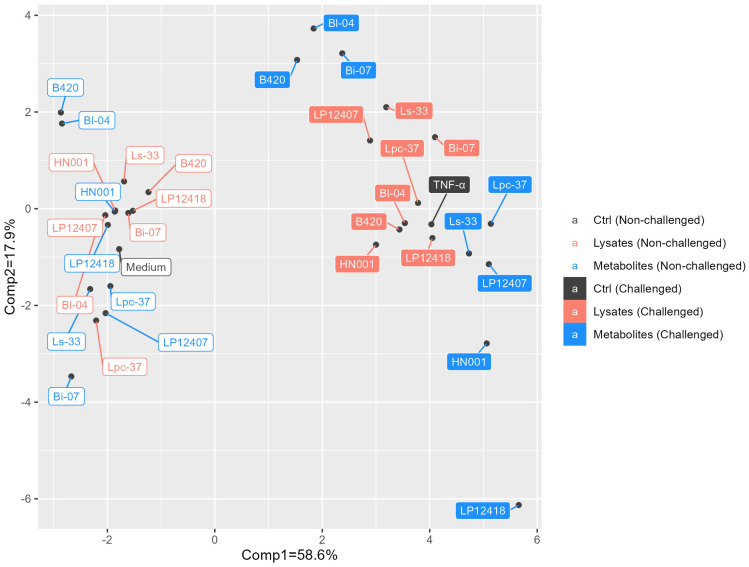
Principal component analysis of the experimental dataset with the first two principal components. Unchallenged samples are indicated by open boxes, and challenged samples are indicated by filled boxes. Different colors refer to different sample types: black—controls, red—lysates and blue—metabolites. Comp1: principal component 1; comp2: principal component 2; ctrl: control.

As a result, 76.5% of the total variation was explained by the first two principal components (Figure 1), wherein the first principal component on the x-axis explained 58.6% of the variation and separated the experimental data into unchallenged and TNF-α-challenged samples. Thus, the TNF-α challenge effected much of the variation in the entire dataset. Regarding the second principal component, explaining 17.9% of the variation in the dataset, the effects were more expansive in challenged samples due to the TNF-α challenge compared with unchallenged samples; in particular, the metabolite samples were more widespread, with bifidobacterial metabolites on the positive side of the second principal component and lactobacilli metabolites on the negative side (Figure 1).

TNF-α challenge increased HDF viability to 136% (unchallenged HDF viability was assigned 100%). Furthermore, TNF-α was not cytotoxic to HDFs but was sufficiently potent to induce metabolic changes. TNF-α challenge increased cell confluence, the MMP-1/type I pro-collagen ratio, and the levels of IL-10, IL-1α, IL-1β, IL-6, IL-8, KGF, TNF-α, and VEGF (Table 1), but decreased type I pro-collagen, FGFb, and TGF-β1.

### 3.2. Effect Differences between Strains and Species, Lysates, and Metabolites on HDF Biomarkers

**Figure 2:**
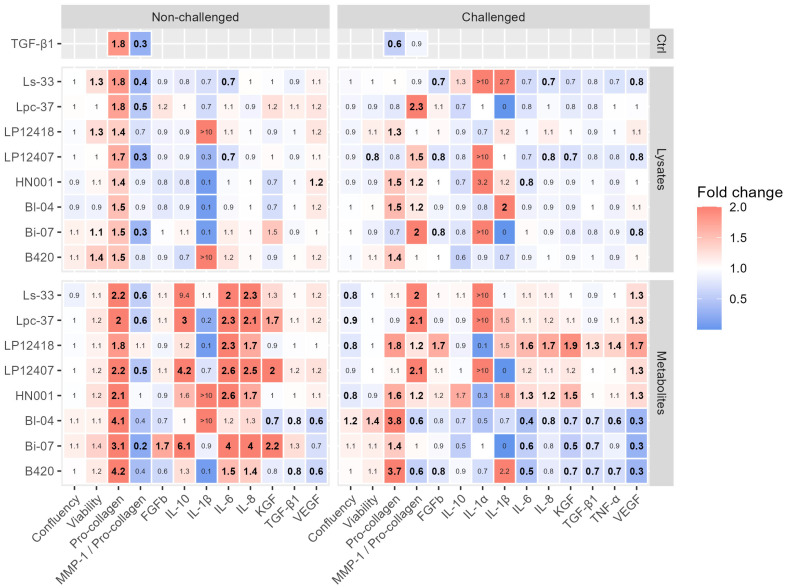
Relative fold-changes in parameters compared with the respective cell passage controls under unchallenged and TNF-α-challenged conditions in HDFs treated with bacterial lysates or metabolites. The controls were medium for the no-challenge data and TNF-α for the challenge data. TGF-β1 was used as a positive control for type I pro-collagen production. Statistically significant (*p* < 0.05) changes versus the respective controls are denoted in bold (detailed *p*-values of the fold-changes can be found in Supplementary Figure S1). Without the challenge, there were no detectable amounts of IL-1α or TNF-α in the samples; thus, the effect sizes are not included in the figure. Ctrl: control.

Next, we examined how the lysed probiotics and their produced metabolites affected HDF viability, confluence, MMP-1/type I pro-collagen ratio, and levels of type I pro-collagen, cytokines, and growth factors (Figure 2).

#### 3.2.1. HDF Viability and Confluence

Viability was not negatively affected by the bacterial treatments, all of which were well tolerated by the HFDs, with no cytotoxicity (Figure 2). Four bacterial lysates increased the viability of unchallenged cells: Ls-33 (1.3-fold, *p* = 0.017), LP12418 (1.3-fold, *p* = 0.031), Bi-07 (1.1-fold, *p* = 0.023), and B420 (1.4-fold, *p* = 0.0027); LP12407 decreased challenged HDF viability 0.8-fold (*p*= 0.0089). No metabolite had significant effects on cell viability without the challenge; under the challenge, only Bl-04 metabolites improved HDF viability (1.4-fold compared with the control, *p* = 0.00019). Confluence was unaffected by any of the lysates, whereas, of the metabolites increased confluence was noted only with Bl-04 (1.2-fold, *p* = 0.0029), versus decreases with HN001 (0.8-fold, *p* = 0.020), LP12418 (0.8-fold, *p* = 0.00077), Lpc-37 (0.9-fold, *p* = 0.017), and Ls-33 (0.8-fold, *p* = 0.012) in TNF-α-challenged cells.

#### 3.2.2. Type I Pro-Collagen and MMP-1/Type I Pro-Collagen Ratio

**Figure 3:**
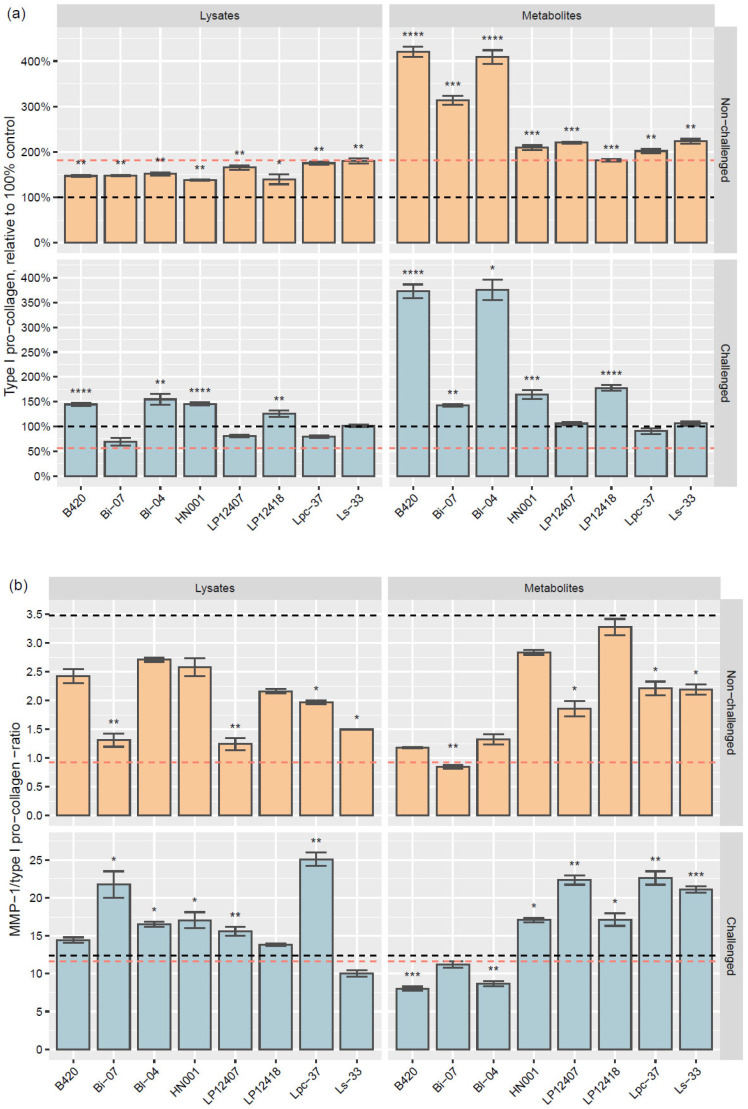
Effects of bacterial lysates and metabolites on (**a**) type I pro-collagen and (**b**) MMP-1/type I pro-collagen ratio in unchallenged and TNF-α-challenged HDF cultures. In (**a**), the values are relative to controls, normalized to 100%. The average of the control levels (medium controls with no challenge and TNF-α controls with challenge, from two datasets of HDF passages) is shown as a black dashed line, and the TGF-β1 positive control for type I pro-collagen production is shown as a red dashed line. The bar graphs show the mean ± standard error (SE), and statistically significant differences between samples and controls (sample compared with the respective HDF passage control) are denoted by asterisks (*) as follows: * *p* < 0.05, ** *p* < 0.01, *** *p* < 0.001, and **** *p* < 0.0001.

All lysates and metabolites increased type I pro-collagen levels in the absence of a challenge, whereas, in challenged cells, Bi-07 lysate and the metabolites and lysates of LP12407, Lpc-37, and Ls-33 did not affect the pro-collagen content (Figure 2). TGF-β1, a positive control for type I pro-collagen production, increased type I pro-collagen 1.8-fold (*p* = 0.016) versus the unchallenged control. The most substantial increases in type I pro-collagen were induced by *B. lactis* B420 and Bl-04 metabolites under both no-challenge and challenge conditions (fold-changes over 3.7, others *p* < 0.0001, except *p* = 0.021 for Bl-04 metabolites under challenge, compared to controls). The metabolite-induced increases were especially notable as bar graphs, relative to the control levels (Figure 3a). The metabolites of *B. lactis* Bi-07 increased pro-collagen levels in unchallenged cells (3.1-fold, *p* = 0.00068) but had more moderate effects after challenge with TNF-α (1.4-fold, *p* = 0.0099), compared with the respective controls.

Under no-challenge conditions, B420, Bl-04, and Bi-07 lysates had a moderate effect on pro-collagen (1.5-fold, *p* = 0.0021; 1.5-fold, *p* = 0.0014; 1.5-fold, *p* = 0.0065, respectively) (Figure 2), as well as in challenged cells (1.4-fold, *p* < 0.0001; 1.5-fold, *p* = 0.0016; and 0.7-fold, not significant). None of the lysates or metabolites decreased pro-collagen levels compared with the control, regardless of challenge. The TGF-β1 control downregulated pro-collagen under the challenge conditions to 60% (0.6-fold, *p* = 0.011) of the TNF-α control (Figure 3a).

The balance between degradation and production is vital in collagen homeostasis; thus, we determined the ratio of MMP-1 produced to type I pro-collagen level (Figure 3b). Under no-challenge conditions, the TGF-β1 control yielded a more balanced MMP-1/pro-collagen ratio of 0.9 (fold-change of 0.3 vs. control, *p* = 0.0023) than the medium control (ratio of 3.5). The no-challenge TGF-β1 control ratio value of 0.9 was considered optimal for this assay. Under the TNF-α challenge, the TNF-α control generated an MMP-1/pro-collagen ratio of 12.4 versus 11.6 with TGF-β1 (not statistically significant).

Under the no-challenge conditions, the MMP-1/type I pro-collagen ratios that were effected by Ls-33, Lpc-37, LP12407, and Bi-07 lysates and metabolites were significantly lower than those by the no-challenge medium control (ratios of 1.2–2.0 and *p*-values of 0.020–0.0090 vs. 0.9–2.2 and 0.031–0.0052, respectively) (Figure 3b). Under the TNF-α challenge, the MMP-1/pro-collagen ratio was best maintained with B420 (ratio 8.0, *p* = 0.00065) and Bl-04 metabolites (ratio 8.7, *p* = 0.0011), compared with the TNF-α control. The highest MMP-1/type I pro-collagen ratio was induced by Lpc-37 lysates under challenge conditions (ratio 25.1, *p* = 0.0013).

#### 3.2.3. Cytokine Levels

There were species-specific and strain-specific differences in the cytokine responses that were elicited by probiotic lysates and metabolites. In general, metabolites had greater effects than lysates in unchallenged and TNF-α-challenged HDFs (Figure 2). IL-6 and IL-8 were increased by nearly all metabolites, except Bl-04, in unchallenged HDFs; IL-6 was decreased by lysates LP12407 (0.7-fold, *p* = 0.0053) and Ls-33 (0.7-fold, *p* = 0.0014). In TNF-α-challenged HDFs, the IL-6 response was mixed, with *B. lactis* metabolites downregulating it compared with control (Bl-04: 0.4-fold, *p* < 0.0001; Bi-07: 0.6-fold, *p* = 0.026; B420: 0.5-fold, *p* < 0.0001) and some lactobacilli increasing it (LP12418: 1.6-fold, *p* = 0.0024; HN001: 1.3-fold, *p* = 0.0027). With lysates, only HN001 elicited a reduction in IL-6 in challenged HDFs. In TNF-α-challenged HDFs, the IL-8 response varied, declining with certain metabolites (Bl-04: 0.8-fold, *p* = 0.027) and lysates (LP12407: 0.8-fold, *p* = 0.029; Ls-33: 0.7-fold, *p* = 0.025) and rising with metabolites of LP12418 (1.7-fold, *p* = 0.0032) and HN001 (1.2-fold, *p* = 0.033).

IL-1β and IL-10 were less affected by the treatments: IL-10 increased significantly only with metabolites that were produced by Bi-07 (6.1-fold, *p* = 0.048), LP12407 (4.2-fold, *p* = 0.0031), and Lpc-37 (3.0-fold, *p*= 0.023) in unchallenged cells, while IL-β rose with Bl-04 lysate 2.0-fold (*p* = 0.043) in TNF-α-challenged cells. IL-1α was undetected in unchallenged samples and unaffected by any treatment in TNF-α-challenged cells.

TGF-β1 and TNF-α experienced minor changes due to the treatments. In unchallenged HDFs, B420 and Bl-04 metabolites decreased TGF-β1 0.8-fold (*p* = 0.021 and *p* = 0.0047, respectively); in TNF-α-challenged cells, these metabolites and those of Bi-07 downregulated TGF-β1 (0.7-fold for all; *p* = 0.0020, *p* = 0.0039, and *p* = 0.045, respectively). The only treatment that increased TGF-β1 was LP12418 metabolites (1.3-fold, *p* = 0.0048). TNF-α levels decreased only with bifidobacterial metabolites B420 (0.7-fold, *p* = 0.0047) and Bl-04 (0.6-fold, *p* = 0.0022) in TNF-α-challenged cells. The only metabolite that increased TNF-α was LP12418 (1.4-fold, *p* = 0.0050).

#### 3.2.4. Growth Factors

Like cytokines, the responses that were elicited by probiotics on growth factors depended on the form of the treatment, for which there were species-dependent and strain-dependent effects. Growth factors generally lacked a response under no-challenge and challenge conditions when HDFs were treated with bacterial lysates. In TNF-α-challenged HDFs, VEGF was decreased by Bi-07, LP12407 and Ls-33 lysates. The only lysate that increased VEGF under no-challenge conditions was HN001, upregulating it 1.2-fold (*p* = 0.034). No lysate affected KGF in unchallenged cells; in challenged cells, only LP12407 lysate had an effect, decreasing it (0.7-fold, *p* = 0.017).

Metabolites had more robust effects on growth factors than lysates. In unchallenged cells, only Bi-07 metabolites increased FGFb (1.7-fold, *p* = 0.0027), whereas, in TNF-α-challenged cells, it rose 1.7-fold with LP12418 metabolites (*p* = 0.0030) and decreased 0.8-fold with B420 metabolites (*p* = 0.029). Bifidobacterial B420 and Bl-04 metabolites lowered VEGF content 0.6-fold (*p* = 0.0024 and *p* = 0.0027, respectively) in unchallenged cells, whereas in TNF-α-challenged cells, the decrease in VEGF was notable with all tested bifidobacterial metabolites (all 0.3-fold with B420, Bi-07, and Bl-04; *p* < 0.0001, *p* = 0.00012, and *p* = 0.00014, respectively). On the contrary, metabolites from lactobacilli increased it, being highest with LP12418 (1.7-fold, *p* = 0.00023).

In unchallenged cells, the effects by metabolites on KGF were mixed, increasing with Bi-07 (2.2-fold, *p* = 0.024), LP12407 (2.0-fold, *p* = 0.0084), and Lpc-37 (1.7-fold, *p* = 0.041) metabolites and decreasing with Bl-04 metabolites (0.7-fold, *p* = 0.044). On the other hand, in TNF-α-challenged HDFs, KGF was downregulated by bifidobacterial metabolites (0.7-fold with B420, *p* = 0.048; 0.5-fold with Bi-07, *p* = 0.0037; 0.7-fold with Bl-04, *p* = 0.034) and increased with LP12418 (1.9-fold, *p* = 0.0024) and HN001 metabolites (1.5-fold; *p* = 0.021).

## 4. Discussion

Collagen is an important structural protein in the skin, with type I collagen, synthesized primarily by dermal fibroblasts, constituting 85% to 90% of dry weight skin. During aging, the production of collagen declines, and its degradation increases, accompanied by decreases in other ECM molecules, including glycosaminoglycans (GAGs), such as hyaluronic acid, and proteoglycans, weakening structural support [1,14,15]. Collagenase MMP-1 degrades fibrillar collagen type I, but the functions of MMPs are nonetheless critical; during wound healing, these proteases alter the wound matrix, facilitating cell migration and tissue remodeling [16]. The structure of the skin maintains its homeostasis when the synthesis and degradation of ECM components are balanced [17]. In this study, we evaluated potential probiotic candidates that support collagen homeostasis by studying bacterial lysates and metabolites in vitro using HDFs during a proinflammatory challenge with TNF-α.

The body responds to sudden negative stimuli with an acute inflammatory response. The term “inflammaging” has been used to describe the chronic, low-grade, underlying inflammation that can result from lifestyle or environmental factors [18,19]. Inflammaging affects the skin structure, inducing collagen and elastin fragmentation and degradation, decreasing the production of new structural proteins, and weakening barrier function [5]. The photo-exposed skin of individuals who are in their 30s already express various markers of inflammaging [20]. UV-B radiation induces TNF-α expression in epidermal keratinocytes and dermal fibroblasts, effecting increases in the secretion of other molecules, including proinflammatory cytokines and chemokines [21].

Stimulation of dermal fibroblasts with TNF-α increases the production of MMP-1 and the proinflammatory cytokines IL-6 and IL-8, as well as degrades type I collagen and decreases its synthesis [22,23]. Furthermore, long-term presence of TNF-α upregulates ROS levels and induces premature senescence in human dermal fibroblasts [6]. Under the no-challenge condition, no extracellular TNF-α was detected after treatment with bacterial lysates or metabolites (Supplementary Figure S4). Challenge with TNF-α increased the production of MMP-1, IL-6, IL-8, IL-10, IL-1α and -β, TNF-α, KGF, and VEGF in control cells and downregulated type I pro-collagen, FGFb, and TGF-β1. Furthermore, it inhibited TGF-β1 control-induced type I pro-collagen synthesis (Figure 2 and Figure 3a). In separate studies with HDFs, a 15 min challenge with TNF-α at 20 ng/mL (comparable concentration with our study) already led to 1.5- to 2.5-fold increases in intracellular ROS and raised phosphorylation of several mitogen-activated protein kinases and nuclear factor kappa B [23,24,25]. Similar effects have been noticed to be the result of UV-B challenge [26]. Thus, we conclude that our challenge was adequate to model the proinflammatory state of the skin and ROS formation, e.g., due to UV radiation, which promotes premature skin aging. Although the 72 h TNF-α challenge did not led to a decrease in cell the proliferation, the possible transition of fibroblasts to the premature senescence state would be interesting to monitor in the future.

Several studies have evaluated the type I pro-collagen production and/or MMP-1 synthesis by HDFs upon exposure to different forms of bacteria, in search of antiaging benefits of bacteria via the gut–skin axis. For instance, the effects of heat-killed bacteria (*Lactobacillus acidophilus* KCCM12625P [27], *Lactiplantibacillus plantarum* HY7714 [28], *Lacticaseibacillus paracasei* MCC1849 [29], and *Lacticaseibacillus rhamnosus* ATCC 7469 [30]) or bacterial components and metabolites (lipoteichoic acid of *Latilactobacillus sakei* KCCM 11175P [31], exopolysaccharide of *L. plantarum* HY7714 [32], and cell-free supernatants of *Bifidobacterium animalis* ssp. *lactis* MG741 [33] and several *Lactobacillus*, *Streptococcus*, and *Bifidobacterium* strains [34]) have been explored. UV radiation activates several signaling pathways, generating interest in the effects of bacterial forms on kinases that influence collagen synthesis and MMP production [28].

In our screen, all bacterial lysates and metabolites increased type I pro-collagen over the medium-only control, with metabolites also exceeding the level of the TGF-β1 positive control. Type I pro-collagen levels were increased most distinctly by *B. lactis* B420 and Bl-04 metabolites, with and without the TNF-α challenge. However, we did not study further for the collagen fibril or fiber formation. There was also less MMP-1 relative to type I pro-collagen with these metabolites during the challenge, indicating a beneficial antiaging effect. A recent systematic review and meta-analysis of probiotic studies for skin photo-aging, including UV challenge, summarized from two murine and four in vitro studies that probiotic stimulation (tested as tyndallized, heat-treated, or fermented plant extracts in the original publications) downregulated MMP mRNA and protein levels [35].

We also examined the effects of bacterial lysates and metabolites on collagen homeostasis, based on HDF confluence and the levels of several cytokines and growth factors. These have been screened less frequently on this scale in vitro with bacterial lysates and metabolites for collagen homeostasis benefits.

In our experiments, Bl-04 metabolites were the only treatment that altered fibroblast cell confluence, increasing it 1.2-fold and improving viability 1.4-fold (Figure 2) compared with the control during the TNF-α challenge. Greater confluence indicates that fibroblast numbers have increased, spread more, or adhered better to the substratum, or that their volume has increased [36]. Varani and colleagues reported that cells in the dermal layer of the skin in older individuals (aged 80+ years) spread less and had a smaller surface area compared with younger people (aged 18–29 years) [14]. They concluded that in chronologically aged skin, fibroblast senescence and the reduction in mechanical tension are significant factors leading to a decrease in collagen synthetic activity, whereas, in photodamaged skin, the loss of tension due to cell contact with fragmented collagen fibers has the largest role, culminating in a decline in collagen synthesis [14,37]. The proliferation, adherence, and spreading of HDFs that were promoted by Bl-04 metabolites might also be factors supporting type I pro-collagen synthesis. The effects of probiotics on the HDF spread, contact with collagen fibers, and mechanical tension of the dermis merit further study but would require a different in vitro model, i.e., a three-dimensional environment.

In this screening dataset, the levels of TNF-α, IL-6, IL-8, IL-10, VEGF, and KGF and the MMP-1/type I pro-collagen ratio were all positively correlated with each other and negatively with type I pro-collagen (Supplementary Figures S2 and S3). None of the strains or forms induced TNF-α production in HDFs without the challenge (Supplementary Figure S4). Under the TNF-α challenge, the bacterial lysates had no influence on TNF-α compared with control, and only metabolites from B420 and Bl-04 decreased its levels; an increase was noted only with LP12418 metabolites.

In addition to stimulating MMP-1 production, TNF-α promotes the release of IL-6 and IL-8, which in turn further upregulates collagen-degrading proteases through the induction of c-Jun N-terminal kinase (JNK) and activator protein-1 (AP-1) [38]. Although Bi-07 metabolites significantly induced IL-6 and IL-8 without the challenge (397% and 405%, respectively, vs. 100% control; Supplementary Figures S5 and S6), it did not increase the MMP-1/type I pro-collagen ratio, although less type I pro-collagen was upregulated with Bi-07 than with B420 and Bl-04 metabolites, which did not induce such increases in IL-6 or IL-8. Under the TNF-α challenge, metabolites of all three *B. lactis* strains effected a 0.4–0.6-fold decrease in IL-6 versus control, representing the only metabolite samples to downregulate IL-6. Only Bl-04 metabolites decreased IL-8 during the challenge and did not induce IL-8 without the challenge.

VEGF has at least six isoforms, and the ELISA that we used detects the VEGF_165_ isoform, which is commonly found in tissues. We noted decreases in VEGF, KGF, and TGF-β1 with *B. lactis* metabolites and a slight decline in FGFb with Bi-07 metabolites under the challenge. In contrast, all lactobacilli metabolites upregulated VEGF, and LP12418 metabolites also increased KGF and FGFb during the challenge. Expression of VEGF_165_ and FGFb induces angiogenesis, especially in wound healing [39,40]. KGF is also expressed by fibroblasts during wound healing, but it specifically stimulates keratinocytes to form a new epithelium [41]. In our study, *B. lactis* metabolites significantly increased type I pro-collagen levels, even under the challenge; thus, the simultaneous declines in VEGF, TGF-β1, and FGFb due to the metabolites, may indicate that fibroblasts slow down the synthesis of these growth factors, which also activate collagen synthesis [42,43,44]. Angiogenesis is stimulated during inflammation and chronic UV exposure [45,46]; while bifidobacterial metabolites downregulated proinflammatory cytokine levels under the challenge, VEGF levels also decreased, which could imply protective effects against chronic photodamage-induced angiogenesis [46]. Metabolites of LP12418 would merit a further study of their wound-healing benefits, given that they increased type I pro-collagen, FGFb, KGF, VEGF, TGF-β1, IL-6, IL-8, and TNF-α during the challenge, all of which are critical in proper wound healing [47].

Probiotics have species-specific and strain-specific differences that can determine their applicability for a certain use [48]. The genetic makeup of a particular strain of *Lactobacillus* spp. or *Bifidobacterium* spp. governs the mechanism via which a probiotic functions in response to a specific environmental condition or stressor [49], and it may largely explain the disparate results between lactobacilli and bifidobacteria. Modes of action for probiotics include colonization and normalization of dysbiotic intestinal microbial communities, competitive exclusion of pathogens and bacteriocin production, modulation of fecal enzymatic activities that are associated with the metabolization of biliary salts and inactivation of carcinogens and other xenobiotics, production of organic acids, cell adhesion and mucin production, and modulation of the immune system [50], each of which can be unique to certain strains or species. Furthermore, the mode of application affects the response, as shown by the bifidobacterial metabolites and lysates in this study and in epidermal keratinocytes by intact bacterial cells, lysates, and probiotic-derived metabolites in our earlier report [11].

Several lactobacilli and bifidobacteria molecules might have dermal effects [51]. In our study, many of the responses were elicited primarily by metabolites or extracellular components that were secreted by probiotics (Figure 2). The metabolites produced by bacteria include organic acids, short-chain fatty acids, and branched-chain fatty acids, as well as secretory proteins, indoles, extracellular vesicles, and bacteriocins [52]. Furthermore, the compositions of the bacterial metabolites can differ, depending on the nutrients and conditions in the intestine and laboratory culture.

Conversely, lysates include surface components of probiotics, such as flagella, pili, surface layer proteins, capsular polysaccharides, lipoteichoic acid, and lipopolysaccharide, as well as intracellular components [52]. Many of these components in lysates are microbe-associated molecular patterns (MAMPs) that interact with immune cells, such as dendritic cells, which might explain the lack of a response by fibroblasts. Fibroblasts are considered part of the “nonclassical” branch of the innate immune system that interacts with immune cells, for instance, during injury repair [53,54]; in our case, we did not have immune cells in our fibroblast culture model. However, we noted differential production of cytokines during various treatments, again showing species specificity; bifidobacterial metabolites reduced IL-6 in the TNF-α challenge, whereas the metabolites of some lactobacilli increased it, as observed with IL-8. In contrast, lysates had little influence on cytokine levels.

In our study, with unchallenged HDFs, bacterial metabolites increased pro-collagen production more than the bacterial lysates, compared with the control. By supporting collagen homeostasis and attenuating the proinflammatory state in dermal fibroblasts, Bl-04 and B420 were the most promising strains for skin antiaging. The effects of oral Bl-04 supplementation on skin health were examined further in a recent clinical intervention trial. Measured by 3D imaging, beneficial effects on several wrinkle parameter were noted with Bl-04 consumption compared with placebo at Week 4 [55]. We did not characterize the lysates or metabolites, but comparing the compositions of *B. lactis* B420, Bi-07, and Bl-04 metabolites could provide insights into the molecules that mediated the favorable effects of Bl-04 and B420 metabolites. Strains B420, Bi-07, and Bl-04 are genetically similar but bear small genomic differences from each other [56,57,58].

## 5. Conclusions

We screened eight probiotic strains, as lysates or metabolites that were produced by bacteria, with regard to their antiaging properties in HDFs in the presence or absence of challenge with TNF-α to model inflammatory damage to the skin structure. There were substantial differences in HDF responses between bacterial species and strains; in general, metabolites elicited more changes in the measured parameters than lysates. The results of the in vitro screen indicate that probiotic Bl-04 metabolites, in particular, promote dermal fibroblast cell proliferation and confluence, and balance collagen homeostasis and immunological status during inflammatory stress, which could have beneficial antiaging effects on the skin structure.

## Figures and Tables

**Table 1:** Effects of tumor necrosis factor alpha (TNF-α) on the human dermal fibroblast (HDF) viability and confluence and levels of pro-collagen, matrix metalloproteinase-1 (MMP-1), cytokines, and growth factors, shown as changes compared with unchallenged control.

Parameter	Effect of TNF-α Challenge on Parameter
Viability	+
Confluence	+
Type I pro-collagen	−
MMP-1/type I pro-collagen ratio	+
FGFb	−
IL-10	+
IL-1α	+
IL-1β	+
IL-6	+
IL-8	+
KGF	+
TGF-β1	−
TNF-α	+
VEGF	+

FGFb: fibroblast growth factor basic, IL-10: interleukin 10, IL-1α: interleukin 1 alpha, IL-1β: interleukin 1 beta, IL-6: interleukin 6, IL-8: interleukin 8, KGF: keratinocyte growth factor, TGF-β1: transforming growth factor beta 1, VEGF: vascular endothelial growth factor. − refers to a decrease, and + denotes an increase compared with unchallenged control value.

## Data Availability

Not applicable.

## Supplementary Materials

The following supporting information can be downloaded at https://www.mdpi.com/article/10.3390/microorganisms11061465/s1: Figure S1. Adjusted *p*-values of relative fold-changes of bacterial lysates and metabolites in human dermal fibroblast (HDF) parameters compared with controls under no-challenge and tumor necrosis factor (TNF)-α challenge conditions; Figure S2. Correlation matrix of measured parameters; Figure S3. Adjusted *p*-values of correlations between parameters; Figure S4. Effects of bacterial lysates and metabolites on TNF-α levels in unchallenged and TNF-α-challenged HDF cultures; Figure S5. Effects of bacterial lysates and metabolites on IL-6 levels in unchallenged and TNF-α-challenged HDF cultures; Figure S6. Effects of bacterial lysates and metabolites on IL-8 levels in unchallenged and TNF-α-challenged HDF cultures.
