# Supplementary material for: Effects of Bacterial Lysates and Metabolites on Collagen Homeostasis in TNF-α-Challenged Human Dermal Fibroblasts
Source: Microorganisms. 2023 May 31;11(6):1465. doi: 10.3390/microorganisms11061465 (PMC10304465; doi:10.3390/microorganisms11061465)
Supplement: Supplementary file 1 [file microorganisms-11-01465-s001.zip › microorganisms-2373911-supplementary.pdf]

Microorganisms

Supplementary Material for Article:

Effects of bacterial lysates and metabolites on collagen homeostasis in TNF-α-challenged human dermal fibroblasts

Laura Huuskonen, Heli Anglenius, Ilmari Ahonen, and Kirsti Tiihonen

Supplementary material

Supplementary Figure S1: Adjusted *p*-values of relative fold-changes of bacterial lysates and metabolites in human dermal fibroblast (HDF) parameters compared with controls under no-challenge and tumor necrosis factor (TNF)-α challenge conditions.
Supplementary Figure S2: Correlation matrix of measured parameters.
Supplementary Figure S3: Adjusted *p*-values of correlations between parameters.
Supplementary Figure S4: Effects of bacterial lysates and metabolites on TNF-α levels in unchallenged and TNF-α-challenged HDF cultures.
Supplementary Figure S5: Effects of bacterial lysates and metabolites on IL-6 levels in unchallenged and TNF-α-challenged HDF cultures.
Supplementary Figure S6: Effects of bacterial lysates and metabolites on IL-8 levels in unchallenged and TNF-α-challenged HDF cultures.

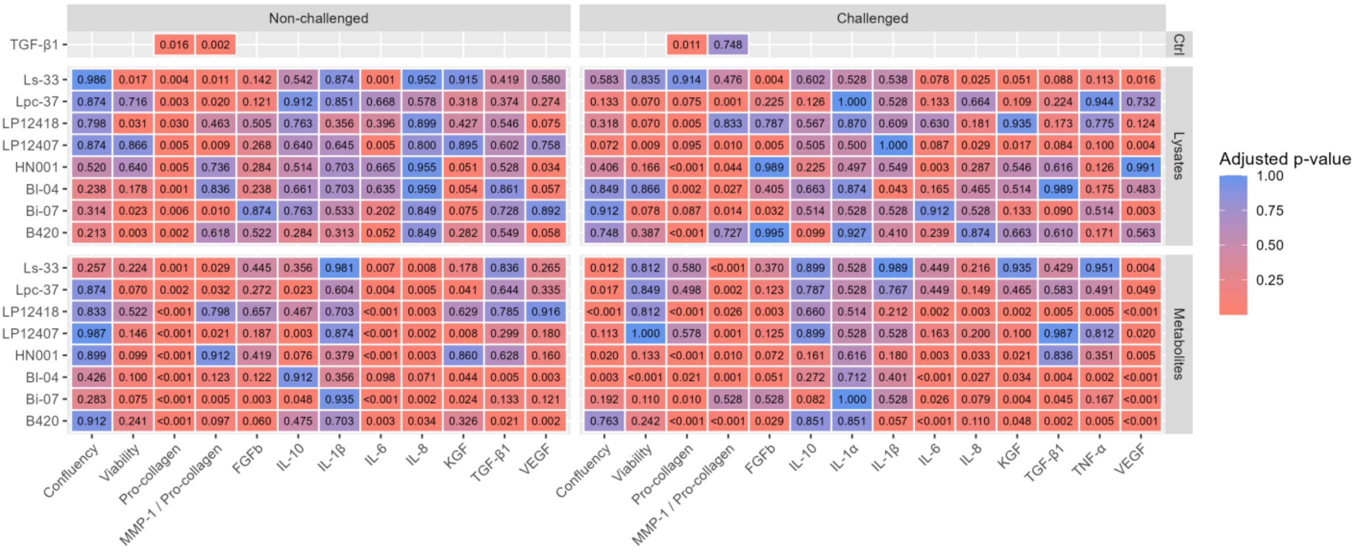

Figure S1. Adjusted *p*-values of relative fold-changes of bacterial lysates and metabolites in human dermal fibroblast (HDF) parameters compared with controls under no-challenge and tumor necrosis factor (TNF)-α challenge conditions. The controls were medium for the non-challenge data and TNF-α for the challenge data. TGF-β1 was used as a positive control for type I pro-collagen production. Without the challenge, there were no detectable amounts of IL-1α or TNF-α in the samples, and thus, the *p*-values are not included in the figure. Ctrl: control, MMP-1: matrix metalloproteinase 1, FGFb: fibroblast growth factor basic, IL-10: interleukin 10, IL-1α: interleukin 1-alpha, IL-1β: interleukin 1-beta, IL-6: interleukin 6, IL-8: interleukin 8, KGF: keratinocyte growth factor, TGF-β1: transforming growth factor beta 1, VEGF: vascular endothelial growth factor.

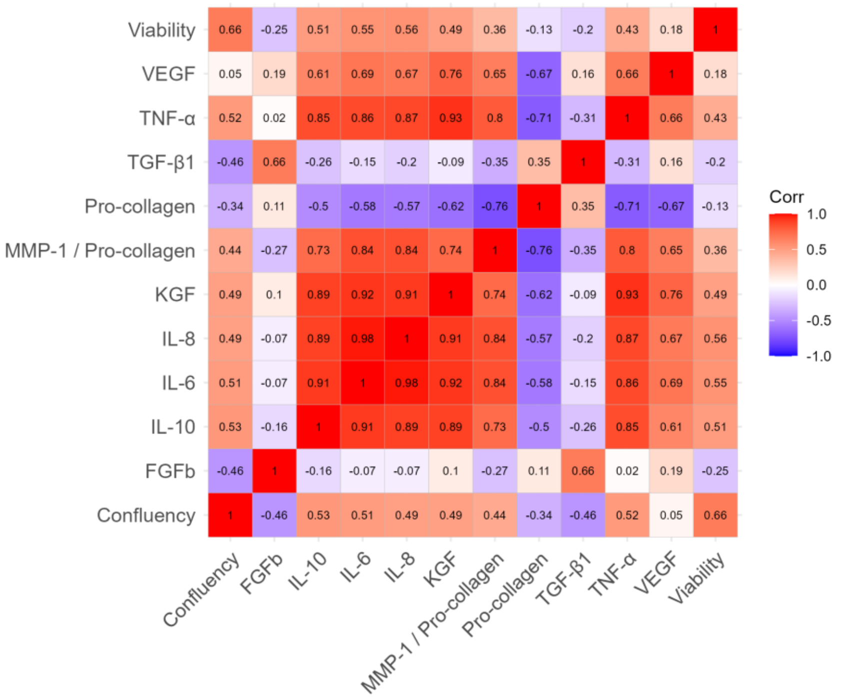

Figure S2. Correlation matrix of measured parameters. The parameters IL-1α and IL-1β were either not detected or were present in low amounts in the samples, and thus, were not included in this correlation analysis. Statistically significant ($p < 0.05$) correlations are denoted in bold (detailed *p*-values of the fold changes can be found in **Supplementary Figure S3**). Corr: correlation.

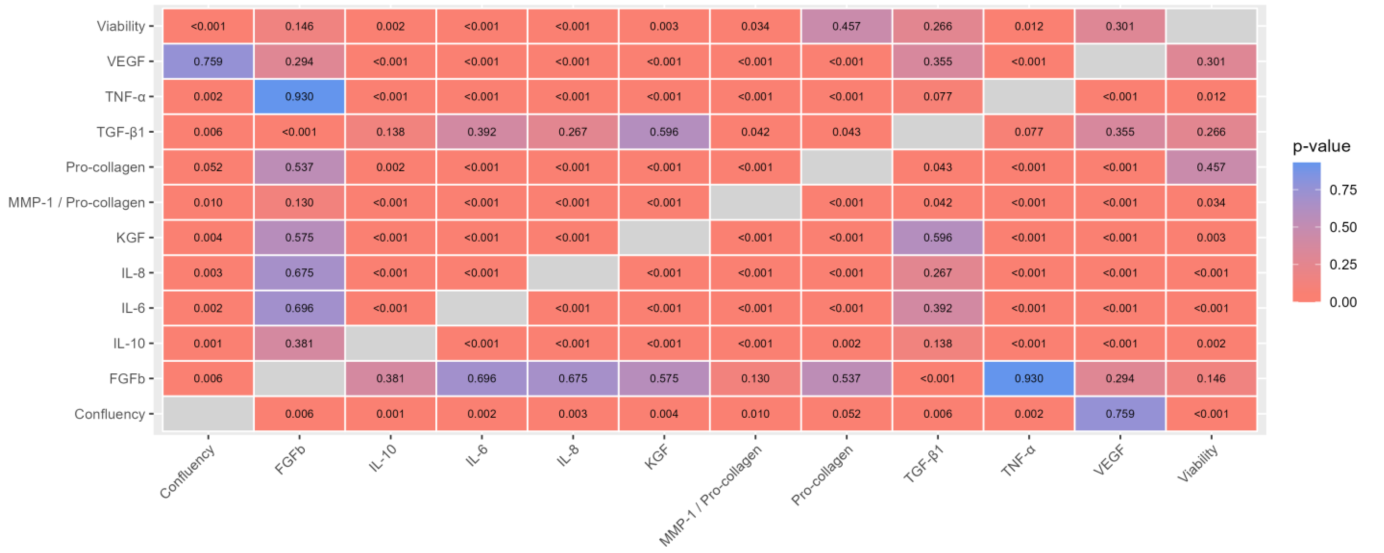

Figure S3. Adjusted *p*-values of correlations between parameters. The parameters IL-1α and IL-1β were either not detected or were present in low amounts, and thus, were not included in the correlation analysis.

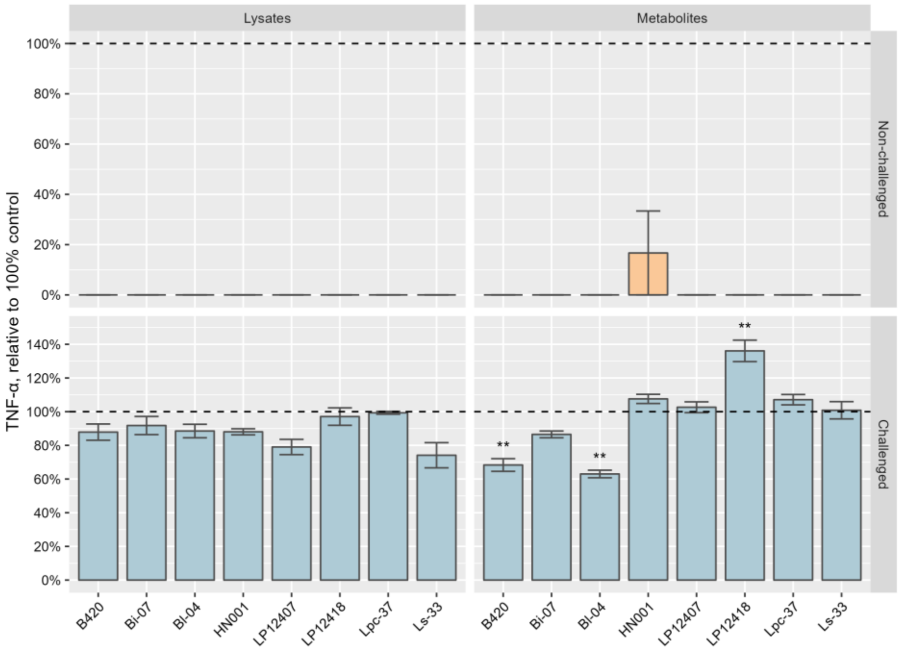

Figure S4. Effects of bacterial lysates and metabolites on TNF-*α* levels in unchallenged and TNF-*α*-challenged HDF cultures. The values are relative to controls, normalized as 100%. Control levels (medium control with non-challenge and TNF-*α* control with challenge) are shown as black dashed lines. The bar graphs show mean ± standard error (SE), and statistically significant differences between samples and controls (sample compared with the respective HDF passage control) are denoted by asterisks (*) as follows: * $p < 0.05$, ** $p < 0.01$, *** $p < 0.001$ and **** $p < 0.0001$.

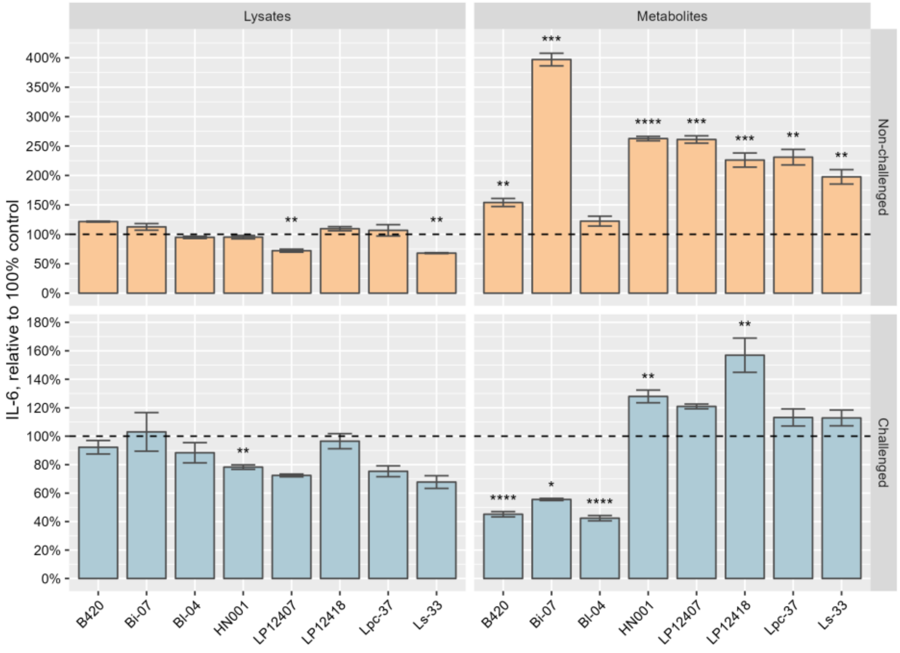

Figure S5. Effects of bacterial lysates and metabolites on IL-6 levels in unchallenged and TNF-*α*-challenged HDF cultures. The values are relative to controls, normalized as 100%. Control levels (media control with non-challenge and TNF-*α* control with challenge) are shown as black dashed lines. The bar graphs show mean ± SE, and statistically significant differences between samples and controls (sample compared with the respective HDF passage control) are denoted by asterisks (*) as follows: * $p < 0.05$, ** $p < 0.01$, *** $p < 0.001$ and **** $p < 0.0001$.

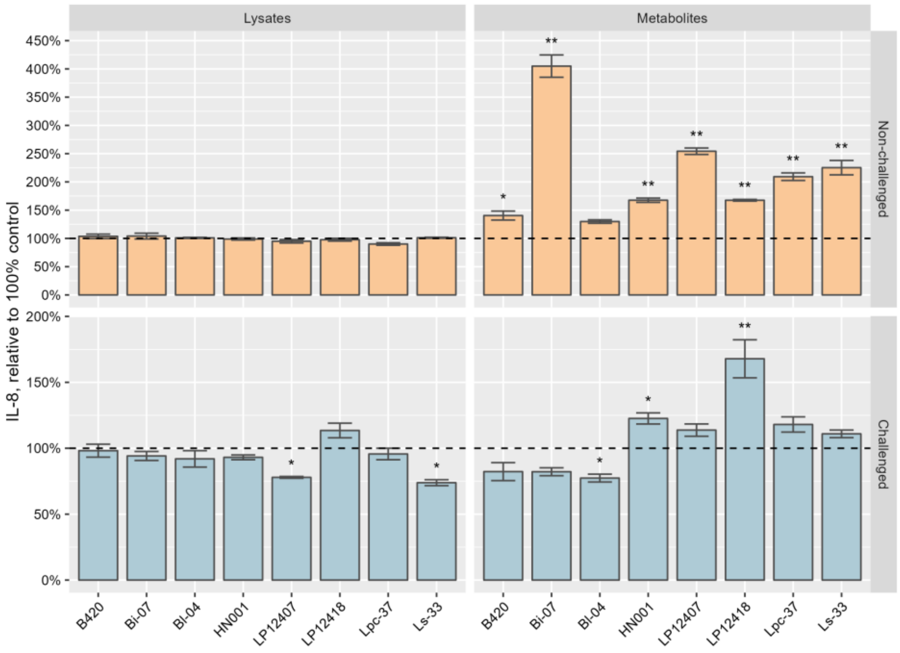

Figure S6. Effects of bacterial lysates and metabolites on IL-8 levels in unchallenged and TNF-α-challenged HDF cultures. The values are relative to controls, normalized as 100%. Control levels (media control with non-challenge and TNF-α control with challenge) are shown as black dashed lines. The bar graphs show mean ± SE, and statistically significant differences between samples and controls (sample compared with the respective HDF passage control) are denoted by asterisks (*) as follows: * $p < 0.05$, **$p < 0.01$, ***$p < 0.001$ and ****$p < 0.0001$.
